# Real-World Impact of GLP-1 Receptor Agonists on Endoscopic Patient Outcomes in an Ambulatory Setting: A Retrospective Study at a Large Tertiary Center

**DOI:** 10.3390/jcm13185403

**Published:** 2024-09-12

**Authors:** Ernesto Robalino Gonzaga, Aimen Farooq, Abdul Mohammed, Saurabh Chandan, Baha Fawwaz, Gurdeep Singh, Amna Malik, Yiyang Zhang, Kambiz Kadkhodayan

**Affiliations:** 1 Department of Gastroenterology, AdventHealth, Orlando, FL 32804, USA; ernesto.robalinogonzaga.md@adventhealth.com (E.R.G.); aimen.farooq.md@adventhealth.com (A.F.); abdulsattarshariq.mohammed.md@adventhealth.com (A.M.); bahaaldeen.banifawwaz.md@adventhealth.com (B.F.); 2 Center for Interventional Endoscopy, AdventHealth, Orlando, FL 32803, USA; kambiz.kadkhodayan.md@adventhealth.com; 3 Department of Internal Medicine, AdventHealth, Orlando, FL 32804, USA; gurdeep.singh.do@adventhealth.com (G.S.); amna.s.malik@adventhealth.com (A.M.); 4 Center for Collaborative Research, AdventHealth Research Institute, Orlando, FL 32804, USA; yiyang.zhang@adventhealth.com

**Keywords:** glucagon-like peptide, anesthesia, sedation, semaglutide, endoscopy, tirzepatide

## Abstract

**Background:** Glucagon-like peptide receptor agonists (GLP-1 RAs) are associated with delayed gastric emptying and may increase the risk of aspiration due to retained gastric contents. There are no guidelines on peri-endoscopic use of GLP-1 RAs, and real-world outcomes in an ambulatory setting remain unknown. This study reports real-world data from an ambulatory center associated with a large tertiary hospital. **Methods:** A retrospective review of electronic medical records was conducted for patients who underwent esophagogastroduodenoscopy (EGD) at a hospital-based outpatient center from January to June 2023. Exclusions included non-elective procedures, current opioid use, altered foregut anatomy, and known gastroparesis. All patients were on GLP-1 RAs before endoscopy and followed standard fasting protocols. Adverse event rates were recorded, and patients were divided into cohorts based on GLP-1 RA use. Univariate and multivariate regression analyses identified risk factors for food retention and complications. **Results:** A total of 1438 patients underwent elective EGD during the study period. Among the 1046 patients included, 73 (7%) were on GLP-1 RAs. The procedure was aborted in four patients (0.4%) due to gastric food retention, with two (50%) on GLP-1 RAs. Independent risk factors for food retention included GLP-1 RA use (OR: 9.19; 95% CI: 2.73–30.8; *p* = 0.0003) and diabetes (OR 5.6; 95% CI: 1.72–18.2; *p* = 0.004). Tirzepatide showed the strongest association (*p* = 0.0056). Factors that did not impact food retention included A1c, BMI, and gender. Protective factors were age (OR 0.96; 95% CI: 0.93–0.99; *p* = 0.02) and same-day colonoscopy (OR 0.18; 95% CI: 0.06–0.58; *p* = 0.003). **Conclusions:** GLP-1 RA use in diabetics increases the risk of retained gastric contents during elective EGD, particularly with tirzepatide, without increasing aspiration risk. Patients undergoing simultaneous colonoscopy had a lower risk of retained gastric contents. Further studies are needed to evaluate the impact of GLP-1 RAs on gastric food retention and procedural risk.

## 1. Introduction

Glucagon-like peptide-1 receptor agonists (GLP-1 RAs) have been used as an anti-diabetic medication since 2005. More recently, they have gained popularity due to their effects on weight management, cardiovascular, and renal benefits [1]. In the metabolic literature, GLP-1 RAs have been linked to delayed gastric emptying [2,3]. These studies have usually used either the 13C breath test [2,4] or the paracetamol absorption technique, which does not necessarily correlate with real-life practice [5,6]. Retrospective studies examining the effect of GLP-1 analogs on increased gastric retention by direct visualization on esophagogastroduodenoscopy (EGD) report conflicting results, likely due to small sample sizes [7,8,9]. Since those studies were published, the use of GLP-1 analogs for diabetes and weight loss has increased exponentially. With GLP-1 RAs gaining popularity, there is growing concern regarding aspiration risk during EGD.

The American Society of Anesthesiologists and the Gastrointestinal Multi-Society statement acknowledge the risks but also highlight the lack of data regarding the complications from aspiration [10,11,12]. The challenge for endoscopists and anesthesiologists is balancing the risk of food retention and aspiration against related complications. Until now, multiple retrospective studies have shown the effect of GLP-1 RAs leading to an increased risk of increased gastric retention [8,13,14,15]. Our study aims to investigate GLP-1 RAs with analysis of real-world data to further determine the risk of food retention and the risk of aspiration during EGD.

## 2. Materials and Methods

Study Population and Data Collection: The study was approved by the institutional review board (IRB) at AdventHealth (Orlando, FL, USA). The electronic endoscopy database (Provation, Minneapolis, MN, USA) was retrospectively queried for elective EGD procedures from 1 January to 30 June 2023, at the outpatient endoscopy center at AdventHealth, Orlando, FL, USA. Exclusions included non-elective procedures, patients under 18, opioid therapy, altered gastric anatomy, known gastroparesis, or obstructive pathologies. All patients followed institutional fasting protocols before the procedure, which included a minimum of 4 h for clear liquids and 6 h for solids. Baseline data included patient demographics, co-morbidities, GLP-1 use, or other medications affecting motility. Procedural data extracted from the endoscopy reports included ASA class, type of procedure, type of anesthesia, presence of food in the stomach, and procedure-related complications.

Outcomes and Definitions: The primary outcome evaluated gastric food retention, defined as any solid food visualized in the esophagus or stomach during esophagogastroduodenoscopy. Secondary outcomes included perioperative broncho-aspiration, endotracheal intubation, aborting endoscopy, and the need for a repeat endoscopy due to gastric retention, hospitalization, and mortality. Factors associated with gastric food retention were then examined.

Statistical Analysis: Univariate logistic regression analyses were performed to assess the individual association between each covariate and the outcome variable of food retention, allowing us to identify variables that have a statistically significant association with the outcome variable at a predetermined significance level (*p* < 0.10). The Hosmer–Lemshow test *p* value is 0.3285, indicating a good model fit. Though the McFadden R^2^ value of our model does not reach 0.4, which is considered an exceptionally good fit, a value of 0.273 still suggests that the model has a reasonably good level of explanatory power. Variables that have a statistically significant association in the univariate analyses were included in the multivariate logistic regression model. Stepwise elimination was then employed to select the final predictor variables. The final multivariate logistic regression model included variables that remained statistically significant (*p* < 0.05) or demonstrated a meaningful impact on the outcome variable after adjusting for other covariates. Patients were divided into two groups: those using GLP-1 agonists and a control group not using these medications. Continuous variables were compared using the *t*-test or Wilcoxon rank sum test. Categorical variables were compared using the Chi-square or Fisher’s exact test.

## 3. Results

**Figure 1:**
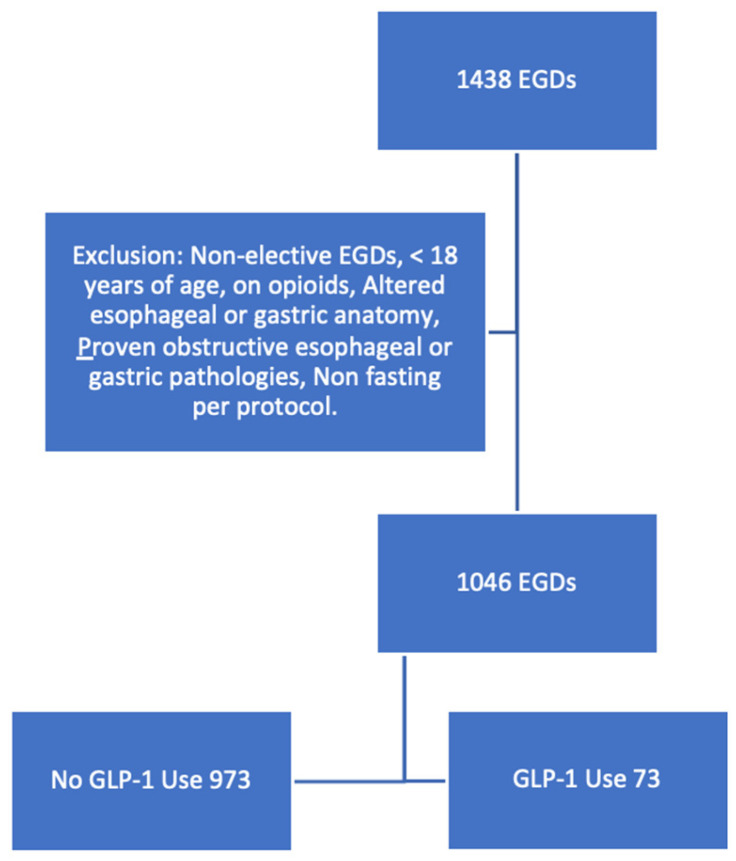
Study design. GLP1-RA, glucagon-like peptide-1–receptor agonist; EGD, esophagogastroduodenoscopy.

A total of 1438 patients underwent esophagogastroduodenoscopy at our outpatient endoscopy suite during the study period. Out of these, 1046 patients (median age 56 years; median BMI 28 kg/m^2^; 64.5% women; 68.6% white) were included in the final analysis as shown in Figure 1. Patient characteristics are summarized in Table 1. The most common comorbidities were diabetes, at 17.6%; followed by coronary artery disease and cirrhosis, each at 10%; and chronic kidney disease at 3.7%. Of the cohort, 73 patients (7%) were using GLP-1 agonists. Among these patients, 39.7% used it for diabetes management, 27.4% for weight loss, and 31.5% for diabetes management and weight loss.

Procedural characteristics are shown in Table 1. All patients included in the final analysis followed institutional fasting protocols. Most patients received monitored anesthesia care (MAC) (*n*; 99.4%). The most common indications for EGD as shown in Table 2 were GERD (32.5%) abdominal pain (24.7%), and dysphagia (15.2%), other indications included nausea, vomiting, bloating, dyspepsia, anemia, esophageal variceal screening, Barrett’s esophagus, abnormal imaging, bariatric surgery pre-operative evaluation, portal hypertension, gastric ulcer, gastrointestinal metaplasia, peptic ulcer disease, esophagitis, odynophagia, rule out GVHD chronic cough, esophageal stenosis, globus sensation, lynch syndrome, gastrointestinal hemorrhage, weight loss, atypical chest pain, esophageal nodule surveillance, FH gastric cancer, familial adenomatous polyposis, history of ampullary adenoma, atrophic gastritis, early satiety, EoE, Crohn’s disease, and celiac disease. A total of 515 patients (49.23%) underwent a colonoscopy on the same day.

**Figure 2:**
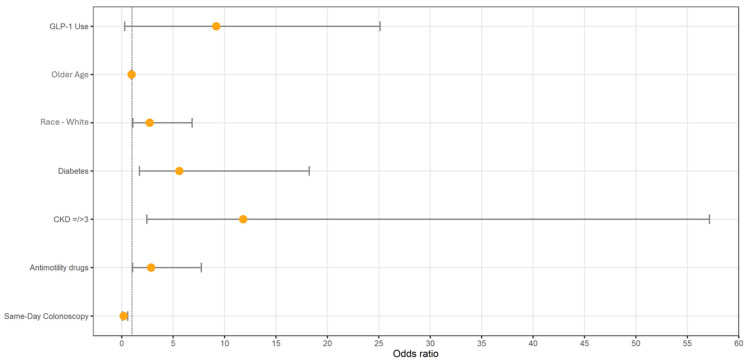
Multivariate analysis for factors that affect the outcome of food retention in patients who underwent upper endoscopy between January and June 2023 (*n* = 1046).

Factors associated with retained gastric contents during esophagogastroduodenoscopy on univariate analysis included history of diabetes (OR 6.3; 95% CI: 2.84–14.27; *p* < 0.005), CKD stage III-IV (OR 5.3; 95% CI: 1.74–16.46; *p* = 0.003), and GLP-1 use (OR 10.1; 95% CI: 4.37–23.47; *p* < 0.005). All these variables remained independent factors associated with a higher likelihood of retained gastric contents visualized during EGD on multivariate analysis. Race, those who identified as white, had a statistically significant predictor of the likelihood of retained gastric products. It is important to note that in this study, this comprised 69% of the population, while the remaining participants represented 1% to 13% of other racial groups. (Figure 2) Undergoing a colonoscopy on the same day appeared to be a protective factor against food retention for those using GLP-1 agonists (OR 0.188; 95% CI: 0.06–0.58; *p* = 0.003). Age also influenced the outcome, where an increase in age (one year) was associated with a 3.71% decrease in the odds of retained gastric content (OR: 0.96; 95% CI: 0.93, 0.99; *p* = 0.023).

Compared to patients not on GLP-1 agonists, patients with GLP-1 agonists had nine times higher odds of food retention (10/73; 13.7% vs. 15/973; 1.54%; *p* < 0.005). Tirzepatide had the highest rate of food retention (5/11; 45.4%), followed by dulaglutide (2/22; 9.1%) and semaglutide (3/37; 8.1%). Exenatide and Liraglutide had too few observations to be included in the analysis. Tirzepatide was associated with a higher risk of food retention than GLP-1 analogs (*p* = 0.005). Among the GLP-1 cohort, patients who underwent endoscopy for abdominal pain, nausea, early satiety, or dyspepsia had no difference in gastric food retention compared to patients undergoing endoscopy for other indications (4/16; 25.0% vs. 10/57; 10.5%; *p* = 0.28). When comparing the odds of food retention in patients with controlled diabetes (HbA1c < 7) to those with uncontrolled diabetes (HbA1c > 7), there was no statistically significant difference among the two groups (6/78; 7.7% vs. 4/44; 9.0%; *p* = 0.79). For more information in regards to independent variable data please refer to Appendix A.

## 4. Discussion

GLP-1 receptor agonists (GLP-1 RAs), the endogenous incretin hormones produced in intestinal endocrine cells, are known for their ability to stimulate insulin release by binding to receptors in the pancreas. Unlike native GLP-1, which has a brief half-life of 2–3 min, GLP-1 RAs exhibit prolonged action, making them valuable for managing diabetes mellitus and promoting weight loss. These agents also reduce glucagon secretion, induce satiety, and delay gastric emptying by binding to gastric neuronal cells, thereby influencing gastrointestinal function [16]. Concerns have been raised regarding the potential for increased risk of retained gastric contents (RGC), and therefore aspiration risk during endoscopy in patients using GLP-1 RAs. There have been case studies reporting instances of gastric content aspiration among patients on GLP-1 RA as they have gained popularity [17,18]. In response, the American Society of Anesthesiologists (ASA) has issued guidelines recommending adjustments in GLP-1 RA administration schedules around procedures, although these recommendations are based on expert opinion rather than robust clinical data [11,19]. Other professional societies, including the American Gastroenterological Association (AGA), have echoed these concerns and emphasized the need for caution during endoscopic procedures in such patients [10].

To aid in a better understanding, our study, which aimed to study real-world data, observed a notable increase (ninefold) in retained gastric contents (RGC) associated with GLP-1 RAs. However, it interestingly did not find a higher risk of aspiration compared to controls. These findings were similar to those by Firkins et al., who, in a cohort of 1512 patients, reported a 2% rate of adverse events with a 0.1% rate of aspiration [14]. Anazco et al., in a larger cohort with 4134 endoscopic procedures, reported pulmonary aspiration in two patients with a rate of 4.8 cases per 10,000 endoscopies [20]. Symptomatic patients receiving GLP-1 RAs, such as those experiencing nausea or vomiting, may benefit from rapid-sequence intubation during endoscopy, as suggested by clinical practice updates from the AGA [12]. However, concordant with the current data, we found no significant difference in RGC between patients undergoing endoscopy for various symptoms with or without GLP-1 RAs, underscoring the complexity of interpreting endoscopic findings in this context.

To explore outcomes among specific GLP-1 RAs, our subgroup analysis revealed varying rates of gastric retention among different agents, with tirzepatide demonstrating significantly higher rates compared to semaglutide or dulaglutide. Tirzepatide’s dual mechanism involving gastric inhibitory polypeptide (GIP), and GLP-1 receptor activation may contribute to this observation, highlighting the need for further mechanistic studies [21]. This was also observed in other studies with tirzepatide having a higher risk of retained gastric contents compared to other GLP-1Ras [14]. Additionally, colonoscopy performed on the same day as GLP-1 RA administration showed lower rates of RGC, possibly due to dietary restrictions the day before the procedure, which reduced bowel residue [21]. Colonoscopy as a potential protecting factor was also found in the study by Firkins et al., with an OR 0.34; 95% CI, 0.23–0.52, *p* < 0.001 [14]. Yao et al. noted that GLP-1RA use was associated with a statistically significant lower quality of bowel preparation in a study involving 446 patients, 59% of whom were GLP-1RA users [21]. Further studies are needed to determine the factors contributing to bowel preparation outcomes in patients using GLP1-RA. While consideration of gastric ultrasound to assess residual contents before anesthesia induction in patients on GLP-1 RAs has been suggested, this lacks robust clinical validation [22]. Balancing potential benefits with logistical challenges and resource allocation remains a consideration for future clinical practice. Further research should explore the utility of this practice in preoperative assessments, in addition to optimizing dietary protocols, to further clarify the clinical implications of adjusting GLP-1 RA therapy around procedures. Additionally, investigating the impact of these adjustments on glycemic control and the necessity for adjunctive diabetes management strategies is warranted.

Our study’s strengths include stringent patient selection criteria excluding those with predisposing factors for RGC other than GLP-1 RA use, adherence to ASA fasting guidelines, and a focus on outpatient settings. Patients in our study therefore met the minimum preoperative fasting standards of 2 h for clear liquids and 6 h for solids. All patients in our analysis underwent an elective endoscopy in the outpatient setting and were not subject to the constraints and risks of hospitalization and urgent endoscopies. Our analysis also has several limitations. First, our study included a relatively small proportion of patients on GLP-I Ras, which may limit the overall strength of our conclusions. Second, it is clear that we have imbalanced data due to the nature of a retrospective single-center study; therefore, variabilities in the outcomes should be expected. However, we believe the findings are clinically relevant and hypothesis-generating since GLP-1 RA use showed a very significant and strong association with the outcome despite the existence of an imbalance. Data collection started in January 2023 at the time when GLP-1RA had an increasing rise in use and stopped in June 2023 due to new institutional regulations implemented after the American Society of Anesthesiologists’ statements concerning the use of GLP-1 receptor agonists peri-endoscopically. These regulations mandate holding the medications one week before procedures or canceling the procedures if this is not performed. Third, there was variability in RGC reporting among endoscopists and we could not quantify the volume of RGC and determine if that affected the AE rate. Fourth, a subset of patients could have undiagnosed gastroparesis. However, we acknowledge that performing a gastric emptying study in all patients on GLP-1 RAs before endoscopy is not practical nor cost-effective.

## 5. Conclusions

Our retrospective analysis demonstrated a higher risk of RGC among patients on GLP-1 RAs prior to endoscopy; however, it did not demonstrate a significantly increased risk of pulmonary aspiration during the procedure. As more clinical data becomes available, we need to weigh the impact of hyperglycemia-related adverse events against the purported risk of pulmonary aspiration in patients on GLP-1 RAs undergoing endoscopy. Further prospective studies can help guide decision-making but will likely be plagued by ethical concerns.

## Figures and Tables

**Table 1:** Demographic characteristics and comorbidities. GLP1-RA indications and endoscopy characteristics. Patients who underwent upper endoscopy between January and June 2023 (*n* = 1046).

Demographics	Entire Cohort (*n* = 1046)	GLP-1 Use (*n* = 73)	No GLP-1 Use (*n* = 973)	*p* Value
Age, median (IQR)	56 (42–66)	57 (47–66)	55 (41–66)	0.1641
Body mass index, median (IQR), kg/m^2^	28 (24.5–32.6)	34.4 (28.6–39)	27.7 (24.4–32.2)	<0.001
Gender %				
Male sex; *n* (%) Female sex; *n* (%)	371 (35.5) 675 (64.5)	21 (28.8) 52 (71)	350 (36) 623 (64)	0.2147 0.2147
Race; *n* (%) White AA/Black Asian American Indian Other	718 (68.6) 132 (12.6) 36 (3.4) 14 (1.4) 141 (13.4)	54 (74) 11 (15) 2 (2.8) 0 5 (6.8)	664 (68) 121 (12.4) 34 (3.5) 14 (1.4) 136 (14)	0.3088 0.5135 0.7333 0.6159 0.0854
Comorbidities %				
Diabetes *n* (%)	184 (17.6)	53 (72.6)	131 (13.5)	<0.001
CAD *n* (%)	105 (10.0)	9 (12.3)	96 (9.9)	0.4995
CHF *n* (%)	23 (2.2)	6 (8.2)	17 (1.8)	<0.001
CKD *n* (%)	39 (3.7)	4 (5.5)	35 (3.6)	0.6182
Severe Pulmonary HTN *n* (%)	2 (0.2)	1 (1.4)	1 (0.1)	0.1348
Liver Cirrhosis *n* (%)	109 (10.4)	17 (23.3)	92 (9.5)	<0.001
GLP-1 AR Use %	7%			
GLP-1 AR for DM	39.7			
GLP-1 AR for weight loss	27.4			
GLP-1 AR for DM & weight loss	31.5			
Anesthesia				
ASA class, *n* (%) I or II III or IV	698 (66.7) 348 (33.3)	24 (32.9) 49 (67.1)	674 (69.3) 299 (30.7)	<0.001 <0.001
MAC sedation %	1040 (99.4)	73 (100.0)	967 (99.4)	0.5010

GLP1-RA, glucagon-like peptide-1–receptor agonist; IQR, interquartile range; AA, African American; CAD, coronary artery disease; CHF, congestive heart failure; HTN, hypertension; DM, diabetes mellitus; ASA, American Society of Anesthesiologists; MAC, monitored anesthesia care.

**Table 2:** Indications for patients who underwent upper endoscopy between January and June 2023 (*n* = 1046).

Indication	Entire Cohort (*n* = 1046)
Abdominal pain; *n* (%)	258 (24.7)
Dyspepsia; *n* (%)	44 (4.2)
Nausea; *n* (%)	29 (2.8)
Vomit; *n* (%)	10 (0.96)
GERD; *n* (%)	340 (32.5)
Dysphagia; *n* (%)	159 (15.2)
Anemia; *n* (%)	53 (5.1)
EV screening/surveillance; *n* (%)	95 (9.1)
Barrett’s esophagus; *n* (%)	33 (3.2)
Abnormal imaging; *n* (%)	16 (1.5)
Bloating; *n* (%)	12 (1.1)

GERD, gastroesophageal reflux disease; EV, esophageal varices.

## Data Availability

Data are stored in a HIPAA-compliant secured location at AdventHealth and can be obtained upon reasonable request.

## Appendix A

**Table A1:** Beta coefficients, Wald statistics (Z value), and beta coefficients for dependent and independent variables.

Dependent Variable	Independent Variable	B (Coefficient)	Exp(B) Odds Ratio (95% CI)	Std. Error	Z Value	Multivariate *p*-Value	Univariate Exp (B) OR (95% CI)	Univariate *p*-Value
Retained gastric products (0 = no, 1 = yes)	Intercept	−1.96541	0.14009848 (0.01153216, 1.70198645)	1.2741	−1.543	0.122933		
	Age	−0.03780	0.9629055 (0.9320168, 0.9948179)	0.0166	−2.272	0.023069 *	0.9806437 (0.9569153, 1.0049604)	0.118
	BMI	−0.03106	0.9694164 (0.9075127, 1.0355427)	0.0337	−0.923	0.356222	0.9994928 (0.9929419, 1.0060870)	0.880
	Sex (male = 0, female = 1)	−0.10730	0.8982596 (0.3446363, 2.3412230)	0.48877	−0.220	0.826243	1.1723024 (0.5010501, 2.7428257)	0.714
	Race (0 = white, 1 = other)	0.99983	2.717820 (1.079320, 6.843701)	0.4712	2.122	0.033841 *	2.0593962 (0.9293269, 4.5636396)	0.0752
	Diabetes (0 = no, 1 = yes)	1.72346	5.603886 (1.722314, 18.233337)	0.6019	2.863	0.004195 **	6.371123 (2.843686, 14.274150)	6.82^−6^ ***
	CAD (0 = no, 1 = yes)	0.03021	1.0306722 (0.1883847, 5.6389162)	0.8671	0.035	0.972206	0.7750106 (0.1801529, 3.3340653)	0.732
	CHF (0 = no, 1 = yes)	0.66559	1.9456377 (0.2184253, 17.3308937)	1.1158	0.597	0.550827	4.1407867 (0.9164373, 18.7095348)	0.0648
	CKD ≥ 3 (0 = no, 1 = yes)	2.46904	11.811079 (2.440936, 57.150859)	0.8044	3.069	0.002146 **	5.365986 (1.748840, 16.464516)	0.00331 **
	Severe pHTN (0 = no, 1 = yes)	−14.20501	6.773938^−7^ (0.00000^0^, Inf)	1024.005	−0.014	0.988932	7.079077^−6^ (0.000000^0^, Inf)	0.991
	Cirrhosis (0 = no, 1 = yes)	0.05398	1.0554638 (0.2609884, 4.2684036)	0.7129	0.076	0.939642	2.2043269 (0.8103285, 5.9964043)	0.122
	GLP-1 use (0 = No, 1 = Yes)	2.21834	9.192094 (2.735788, 30.884924)	0.6184	3.588	0.000334 ***	10.137566 (4.377462, 23.477131)	6.45^−8^ ***
	Prokinetics (0 = no, 1 = yes)	0.98948	2.6898428 (0.2880134, 25.1212462)	1.1399	0.868	0.385385	2.0854167 (0.2687986, 16.1792618)	0.482
	Antimotility drugs (0 = no, 1 = yes)	1.04904	2.854921 (1.053507, 7.736613)	0.5086	2.062	0.039166 *	2.2778465 (0.9922414, 5.2291554)	0.0522
	Same-day colonoscopy (0 = no, 1 = yes)	−1.67033	0.18818435 (0.06086341, 0.58184954)	0.5759	−2.900	0.003728 **	0.25049020 (0.09329786, 0.67252710)	0.00601 **
	ASA (3 and 4 vs. 1 and 2)	−0.78645	0.4554597 (0.1305994, 1.5883954)	0.6374	−1.234	0.217223	1.5947435 (0.7162984, 3.5504852)	0.253

Signif. codes: 0 ‘***’ 0.001 ‘**’ 0.01 ‘*’ 0.05 ‘.’ 0.1 ‘ ’ 1.
